# Corticosteroid Pulses for Hospitalized Patients with COVID-19: Effects on Mortality

**DOI:** 10.1155/2021/6637227

**Published:** 2021-03-12

**Authors:** Ivan Cusacovich, Álvaro Aparisi, Miguel Marcos, Cristina Ybarra-Falcón, Carolina Iglesias-Echevarria, Maria Lopez-Veloso, Julio Barraza-Vengoechea, Carlos Dueñas, Santiago Antonio Juarros Martínez, Beatriz Rodríguez-Alonso, José-Ángel Martín-Oterino, Miguel Montero-Baladia, Leticia Moralejo, David Andaluz-Ojeda, Roberto Gonzalez-Fuentes

**Affiliations:** ^1^Internal Medicine Department, Hospital Clínico Universitario de Valladolid, Spain; ^2^Cardiology Department, Hospital Clínico Universitario, Valladolid, Spain; ^3^Internal Medicine Department, Hospital Universitario de Salamanca-IBSAL-Universidad de Salamanca, Spain; ^4^Internal Medicine Department, Hospital Universitario de Burgos, Spain; ^5^Pulmonology Department, Hospital Clínico Universitario de Valladolid, Spain; ^6^Intensive Care Unit Department, Hospital Universitario de Burgos, Spain; ^7^Intensive Care Unit Department, Hospital Clínico Universitario de Valladolid, Spain

## Abstract

**Objectives:**

To assess the influence of corticosteroid pulses on 60-day mortality in hospitalized patients with severe COVID-19.

**Methods:**

We designed a multicenter retrospective cohort study in three teaching hospitals of Castilla y León, Spain (865,096 people). We selected patients with confirmed COVID-19 and lung involvement with a pO2/FiO2<300, excluding those exposed to immunosuppressors before or during hospitalization, patients terminally ill at admission, or those who died in the first 24 hours. We performed a propensity score matching (PSM) adjusting covariates that modify the probability of being treated. Then, we used a Cox regression model in the PSM group to consider factors affecting mortality.

**Results:**

From 2933 patients, 257 fulfilled the inclusion and exclusion criteria. 124 patients were on corticosteroid pulses (250 mg of methylprednisolone for three days), and 133 were not. 30.3% (37/122) of patients died in the corticosteroid pulse group and 42.9% (57/133) in the nonexposed cohort. These differences (12.6%, 95% CI [8·54-16.65]) were statically significant (log-rank 4.72, *p* = 0, 03). We performed PSM using the exact method. Mortality differences remained in the PSM group (log-rank 5.31, *p* = 0.021) and were still significant after a Cox regression model (HR for corticosteroid pulses 0.561; *p* = 0.039).

**Conclusions:**

This study provides evidence about treatment with corticosteroid pulses in severe COVID-19 that might significantly reduce mortality. Strict inclusion and exclusion criteria with that selection process set a reliable frame to compare mortality in both the exposed and nonexposed groups.

## 1. Introduction

In December 2019, a new betacoronavirus called SARS-CoV-2 induced severe bilateral pneumonia similar to severe acute respiratory syndrome (SARS), described in 2003. This coronavirus disease (COVID-19) had lower mortality than SARS-CoV-1 infection but higher infective capacity. The epidemic began in Wuhan, mainland China, but in a few months became pandemic.

Spain was one of the world's most affected countries, especially in Madrid, Catalonia, and Castilla y León regions [1].

After 32 885 641 cases were confirmed, mortality rates are between 3 and 4% [2], mostly due to acute respiratory distress syndrome (ARDS) and micropulmonary embolism. These symptoms are related to a hyperinflammatory state and a cytokine storm syndrome in some patients [3]. Thus, several authors have postulated that immunosuppressor agents (like corticosteroids, anakinra [4, 5], or tocilizumab [6, 7]) might be useful for these patients.

Several studies have tried corticosteroids for the treatment of viral pneumonia (including Flu and SARS-CoV-1) and ARDS, with different results [8–26]. Only a few studies demonstrate the benefits of corticosteroids on mortality [15, 16, 27, 28]. The Recovery trial's preliminary results obtained mortality benefits with dexamethasone treatment in COVID-19 patients that required oxygen supplementation [29].

Corticosteroids inhibit the migration of leukocytes to inflamed tissues, enhancing their migration from bone marrow to blood [30] and decreasing leukocyte apoptosis [31]. They also inhibit leukocyte reactive oxygen species, increase IL-10 [32, 33], and alter the maturation and differentiation of dendritic cells [34–36]. Corticosteroids modify NK cytolytic activity and monocyte activation [36]. They also downregulate IL-1, IL-2, IL-6, IL-8, IFN-*γ*, or TNF-*α* by transrepression [37].

The dose and the timing of corticosteroids are essential to determine their effect. There are three moments in which the use of corticosteroids might be especially useful. These are the onset of acute lung injury, the initial phase of ARDS, and ARDS refractory to treatment [38].

At thirty to one hundred mg of prednisone equivalent daily dose, corticosteroids act over cytosolic glucocorticoid receptors (cGCR), following the so-called genomic pathway [38, 39]. The genomic pathway effect is highest at 100 mg daily dose. The complex formed by glucocorticoid and its cytosolic GCR has two actions: promotion of anti-inflammatory transcription factors (transactivation) like IL-10 and annexin 1 and inhibition of inflammatory transcription factors (transrepression) like IL-1, IL-2, IL-6, interferon-*γ* (IFN-*γ*), prostaglandins or tumor necrosis factor *α* (TNF-*α*), and IL-8. All these changes carry out from hours to days.

If we use an equivalent dose of prednisone higher than 100 mg daily (so-called pulse corticosteroids), we obtain the maximum effect of the genomic pathway and additional responses from the faster “nongenomic pathway” [37]. These nongenomic mechanisms include membrane dysfunction in all immune cells (including lymphocytes), with a delayed flow across the membrane in the calcium and sodium channels with subsequent decreased ATP production. Other nongenomic effects are binding to membrane GCR in T cells [37] or the release of Src protein from the complex cGCR multiprotein (anti-inflammatory effects). This quick (in hours) and effective action [40] justifies their use in life-threatening situations in autoimmune diseases.

## 2. Methods

We analyzed patients with COVID-19 admitted between March 12th and May 20th to three tertiary teaching hospitals in Castilla y León, Spain: Hospital Clínico Universitario de Valladolid (HCUV), Hospital Universitario de Salamanca (HUSA), and Hospital Universitario de Burgos (HUBU). The three hospitals cover all hospital admissions in a geographical area corresponding to 865 096 people.

The treating team decided on the prescription of all drugs without any intervention from investigators. We obtained the local ethics committee (CEIC) permission to perform the study. Informed consent was obtained. We designed a retrospective cohort study and compared a cohort of patients exposed to corticosteroid pulses and an unexposed one.

### 2.1. Data Source

We analyzed paper and electronic records in all hospitals. We recorded variables related to clinical outcomes and corticosteroids exposure (supplementary material section A (available here)).

### 2.2. Inclusion and Exclusion Criteria

We included patients older than 18 years, testing positive on SARS-CoV-2 PCR (nasopharyngeal or oropharyngeal swab specimens). Patients with positive ELISA serology and consistent clinical symptoms were also considered confirmed cases.

All included patients had a significant lung involvement, defined as a pO2/FiO2<300, maintained for 24 hours or repeated for three days. We measured pO2/FiO2 in arterial gasometry or estimated it from pulse oximetry data (nonlinear estimate model) [41, 42].

We excluded patients receiving classic immunosuppressors or cytokine blockers (as cyclosporine, tocilizumab, or anakinra). Concomitant drugs allowed were hydroxychloroquine, azithromycin, remdesivir, lopinavir/ritonavir, and colchicine. We excluded patients who died in the first 24 hours of admission. Patients on corticosteroid treatment in a different regimen than the one described in this study were excluded. We also excluded pregnant women, terminally ill patients, and patients under a limitation of therapeutic efforts during the first 24 hours of admission.

### 2.3. Corticosteroid Pulse Definition

We considered exposure to corticosteroid pulses if administered at a daily dose of 125 to 500 mg of intravenous methylprednisolone for two to five days. We did not include patients with repeated corticosteroid pulses nor treatments longer than five days. About timing, we considered corticosteroid pulses in the ±3 days, respecting the inclusion criterion date.

### 2.4. Endpoints

The primary endpoint was 60-day mortality in exposed versus nonexposed patients. Secondary endpoints were 30-day mortality, intensive care unit (ICU) admission, in-hospital stay, viral shedding until negative PCR, and serious adverse events, including infections.

### 2.5. Statistical Analysis

We expressed continuous variables with the median and interquartile range (mean and standard deviation if they had normal distribution). We used chi-square to compare qualitative variables and the *t*-test (if normal distribution) or the Mann Withney test to compare two quantitative variables. We performed the Kolmogorov-Smirnov test to examine normal distribution.

We performed a propensity score matching to balance the difference of covariates related to exposure to corticosteroid pulses.

To select suitable covariates to control, we set biologically plausible variables related to the probability of being treated with corticosteroids pulses (propensity score). First, we analyzed variables associated with the propensity score in univariate analysis. Variables found significant were dichotomized, and then, we performed a binary logistic regression to evaluate independent variables associated with the propensity score. We chose three matching methods (propensity score matching) to preprocess the sample: the nearest neighbor, the nearest neighbor with a caliper (at a distance of 0.05, 0.1, 0.2, and 0.3), and exact matching. We performed the propensity score matching using the R software, with the MatchIt and Cobalt libraries.

We checked the balance of the propensity score matching through the “difference of means” to ensure that the distribution of covariates was similar in the treated and control group, and we picked the best-matched model.

Once we completed the propensity score matching, we performed a Cox proportional hazard regression analysis to evaluate mortality and intensive care admission.

## 3. Results

### 3.1. Patients

**Figure 1:**
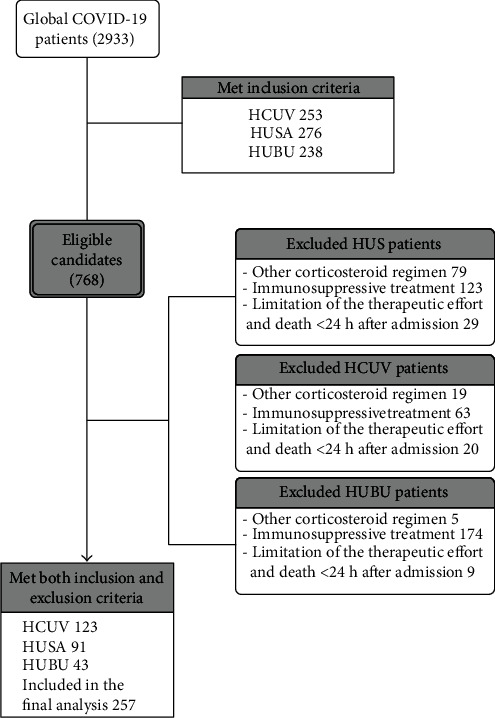
Flow diagram of COVID-19 included patients in this study.

From 2933 patients in our cohort, 257 fulfilled the inclusion and exclusion criteria. 767 fulfilled the inclusion criterion, and 546 had any exclusion criteria (see Figure 1). We diagnosed with COVID-19 in 243 patients based on SARS-CoV-2 PCR in the nasopharyngeal or oropharyngeal swabbing and 14 patients based on positive serology with compatible symptoms. 124 patients were on corticosteroid pulses, and 133 were not. The most used corticosteroid pulses dose was 250 mg daily for three days (92%,114/124).

### 3.2. Propensity Score Matching

We calculated the propensity score (probability of being treated with corticosteroid pulses) in each participant from a binary logistic regression. Variables statically significant in the binary logistic regression were as follows: epidemiological week, presence of bilateral infiltrates or not, Center in Castilla y León, Ferritin, and COVID-gram score (see supplementary material section B (available here)).

After performing the propensity score, we tried several preprocessing methods for matching (see Methods), and we selected the exact matching method as it got the minimum difference between groups with the minimum sample loss (28 controls and 2 treated patients).

**Figure 2:**
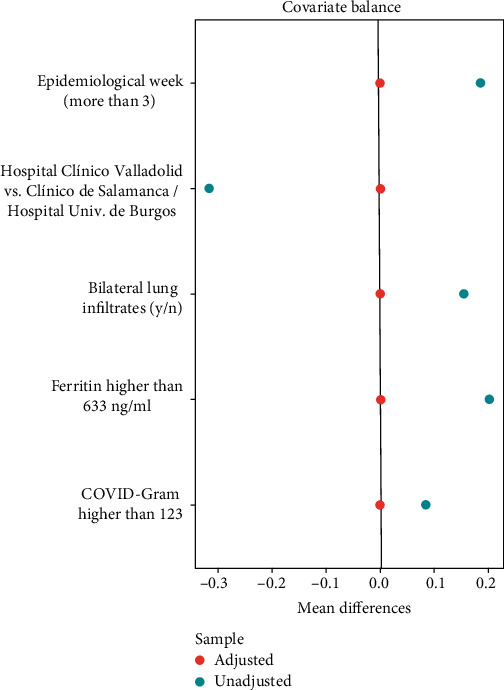
Love plot of the propensity score matching using the exact method.

The difference of means was zero in the treated versus the controls because we used the exact matching method (Figure 2 and supplementary material section B (available here)).

After the propensity score matching, the sample consisted of 207 patients (119 treated and 88 controls).

### 3.3. Baseline Features

The median age in all participants (257) was 75 [63.5-83] years. One hundred and eleven participants (43.2%) were women. Their median classic Charlson score was 1 [0-3].

Comparing patients exposed to corticosteroid pulses and the nonexposed ones, we found that age and comorbidities were similar in both groups, without significant differences. The COVID-gram score [43] was 155.8 in the bolus group and 152.3 in the control group, even decreasing these differences after matching. The classic Charlson score was significantly higher (0.7 points) in the control group (*p* = 0.012). These differences disappeared after dichotomizing the variable in the matched group (≤2 or >2 more than 2 points, *p* = 0.171) (see Table 1).

LDH and ferritin at admission were higher in the pulse group, but these differences disappeared after matching. Peak ferritin and peak LDH during hospitalization were significantly higher in the pulse group, even after matching (see Table 1).

Concomitant treatments with colchicine, interferon beta-1b, lopinavir/ritonavir, and azithromycin were more common in the corticosteroid pulse group than in the controls, both before and after matching (see Table 1).

We did not find differences in pO2/FiO2 between the pulses and control group (*p* = 0.183 in all participants and *p* = 0.69 in the matched group), but bilateral lung infiltrates were more frequent in the corticosteroid pulse group (*p* = 0.006). That difference disappeared after matching (*p* = 0.299).

### 3.4. Outcomes

#### 3.4.1. Primary Endpoint

**Figure 3:**
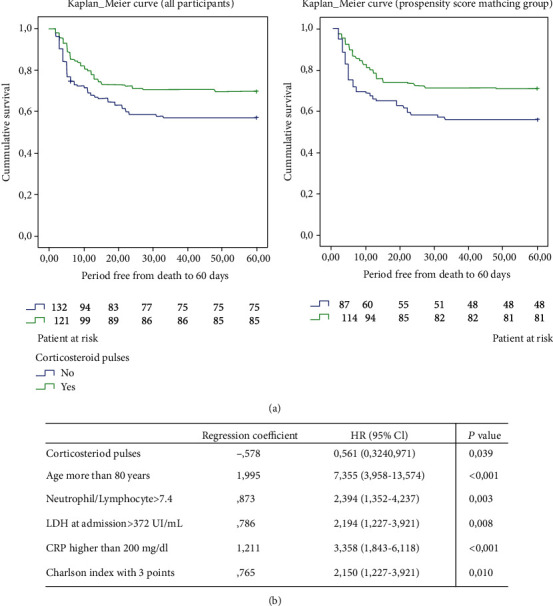
(a) Kaplan-Meier estimates of 60-day mortality in patients with and without corticosteroid treatment. (b) Cox regression for evaluating the 60-day mortality in the matched population.

Ninety-four patients died during the 60 days after admission, representing 36.9% (94/255) of the sample. 30.3% (37/122) of patients died in the corticosteroid pulse group and 42.9% (57/133) in the nonexposed cohort. These differences (12.6%) were statically significant in the Kaplan Meier curve (log-rank 4.72, *p* = 0.03) (see Figure 3).

We carried out a propensity score matching and calculated the mortality in the matched group. The corticosteroid pulse group had a 60 days mortality of 29.6% (34/115), while mortality in the control group was 44.3% (39/88). Again, these differences were statically significant (log-rank 5.31, *p* = 0.021) (see Figure 3 and Table 2).

**Figure 4:**
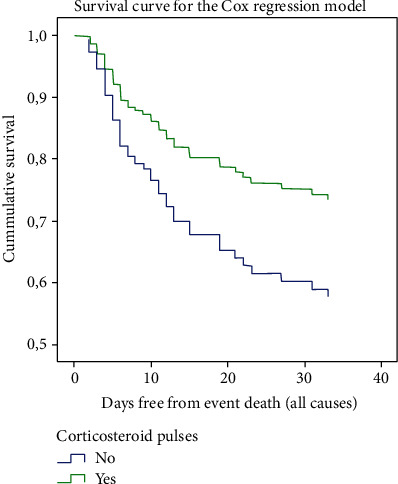
Survival for the Cox regression model in corticosteroids exposed and nonexposed patients (in the PSM group).

We performed a multivariate analysis using a Cox regression model in the propensity score matching group, after dichotomizing variables, and those independently related to 60-day mortality were as follows: corticosteroid pulses (HR 0.561, *p* = 0.039), age older than 80 years (HR 7.3, *p* < 0.001), CPR at admission higher than 200 mg/dL (HR 3.35, *p* < 0.001), neutrophil/lymphocyte index > 7.4 (HR 2.15, *p* = 0.010), Charlson index higher than 2 points (HR 2.15, *p* = 0.018), and LDH at admission > 372 UI/mL (HR 2.29, *p* = 0.008) (see Figure 4). In the equation, we did not include other variables (like pO2/FiO2, lung infiltrates, or D-dimer) that were not significant in the multivariate model, although important in the univariate analysis.

We studied the possible association of colchicine, azithromycin, and lopinavir/ritonavir on mortality, as those treatments were more frequently used in the corticosteroid pulse group. The three treatments were associated with lower mortality in the Kaplan-Meier curves (*p* = 0.008 for colchicine, *p* = 0.032 for lopinavir/ritonavir, and *p* < 0.001 for azithromycin). These differences disappear after adjusting for age higher than 80 years in both three drugs (*p* = 0.1 for colchicine, *p* = 0.794 for lopinavir/ritonavir, and *p* = 0.378 for azithromycin).

Patients on corticosteroid pulses had a higher, but not significant, anticoagulation rate (86.9% vs. 83.9%, *p* = 0.49). Patients on pulses also used significantly lower prophylactic dose heparin and higher intermediate and anticoagulant dose. Still, we did not find in our study an association between the absence of anticoagulation and mortality (log-rank 0.9, *p* = 0.338) (see supplementary material Section D).

#### 3.4.2. Secondary Endpoints

The thirty-day mortality was 30.3% (37/122) in the corticosteroid pulse group and 42.1% (56/133) in the nonexposed cohort. The difference was statically significant (log-rank = 4.3, *p* = 0.038) in the Kaplan-Meier curve. That difference remained in the propensity score matching group (*p* = 0.03) (See supplementary material (available here)). After using the same Cox regression model used in the 60-days mortality analysis, corticosteroid pulses remain a protective factor for 30-days mortality (*p* = 0.049) (see supplementary material section C1 (available here) and Figure 3).

Forty-nine patients were admitted to ICU during this period. ICU admission was 18.5% (23/124) in the corticosteroid pulses cohort and 19.5% (26/133) in the nonexposed group (*p* = 0.838). We found similar results in the propensity score matching group (ICU admission 16.2% in the corticosteroid group and 23.9% in the control group, *p* = 0.173). The time from hospital admission to ICU admission was zero to four days, and 83.6% of patients were admitted in the first 24 hours. Mortality among the ICU admitted patients was 17.4% (4/23) in the exposed group and 61.5% (16/26) in the nonexposed group. This difference was statistically significant for 60-day mortality (log-rank = 9.7, *p* = 0.002). This difference remains in the propensity score-matched group (15.8% vs. 61.9%, *p* = 0.003). After adjusting for several variables (peak LDH, peak CRP, number of comorbidities, D-dimer at admission, SaO2/FiO2, and age) in a Cox regression, those differences in mortality were not significant (see supplementary material section C2 (available here)). The ICU average stay was 19.2 and 21.67 days for both the exposed and nonexposed cohorts, respectively (*p* = 0.750).

The in-hospital median stay was 12 [7.25-19.75] days in the treated cohort and 8 [5–15] days in the nonexposed group (*p* < 0.001). These differences remain in the matched group (*p* = 0.001). However, differences were not statistically significant if we analyzed them in the survivor's group (difference of means 1.2 days, *p* = 0.619).

Viral shedding until negative PCR was shorter (but not significant, *p* = 0.279) in the corticosteroid cohort (25.02 days in the corticosteroid group and 30.65 days in the nonexposed).

We reported serious adverse events in 17 patients in the exposed cohort and 15 patients in the control group (*p* = 0.133). Hemorrhage happened in 9 patients, without difference between groups (*p* = 0.053). In-hospital infections were not higher in the corticosteroid bolus group (29/124) than in the nonexposed cohort (32/129) (*p* = 0.792).

We found 27 patients with pulmonary embolism by CT scan (8 in the exposed group and 19 in nonexposed, *p* = 0.066). We also reported two ischemic strokes and one acute myocardial infarction.

## 4. Discussion

Corticosteroid pulses have been widely used in Spain, especially in the Castilla y León region, but not in other countries to treat COVID-19. The rationale of its use is to stop the systemic inflammation process [3] that develop in some patients with severe COVID-19. Some studies [44–46] have described the positive effects of corticosteroid pulses on mortality in patients with severe COVID-19.

We found a significant improvement in survival in patients treated with corticosteroid pulses. We designed a retrospective cohort study to confirm this statement, which is the main limitation of our work. As there is no randomization, unknown confounders might be unattended.

We carried out a multicenter study with three teaching hospitals in the Castilla y León region in Spain. This fact is one of the main strengths of the study. On the one hand, we summarized various treatment protocols in each center, showing a wider specter of treatment options for severe COVID-19. This protocol variety determines different probabilities of being treated with corticosteroids in each center and enables us to adjust them in the latter propensity score matching. It also considers different hospital admission criteria and different extrahospital resources that may change the baseline features of hospitalized patients.

On the other hand, it represents all hospital admissions in a geographic area in Castilla y León with more than 865 000 people that have similar epidemiological features. They also had the same timing of lockdown and the same mobility restrictions over time.

We used strict inclusion and exclusion criteria to avoid mixing the effects of other immunosuppressive treatments in mortality. They were also useful to find an adequate patient profile who, a priori, should benefit on an anti-inflammatory treatment as corticosteroid pulses. We selected patients, at the inclusion time, with the onset of an acute respiratory distress syndrome.

To limit potential biases, we performed a propensity score matching (PSM) using the exact method. Thus, we obtained a more homogeneous sample with baseline features that were similar in both groups. Both the exposed and nonexposed cohorts in the propensity score matching group had comparable age, a similar pretest probability of dying (through the COVID-gram score), probability of being treated with corticosteroids, and uniform comorbidities. We again found, in this PSM group, the same mortality decrease in the corticosteroid pulse arm. Then, to adjust other possible mortality causes, we performed a Cox regression multivariate model on the PSM group, once more finding a significant protective role of corticosteroid pulses in mortality.

Altogether, joining these strict inclusion and exclusion criteria with all this selection process sets a reliable frame to compare mortality in both the exposed and nonexposed groups. Thus, corticosteroid pulses might be a good option for the treatment of severe COVID-19, as they have been shown to be effective in reducing mortality in our cohort and they are inexpensive and highly available worldwide. Our results can only extrapolate to patients with severe COVID-19, with a pO2/FiO2 lower than 300, not exposed to any kind of immunosuppression, and in the absence of a terminally ill situation at admission.

Some recent studies have confirmed that oral or intravenous low-dose corticosteroids positively affect mortality [29]. There was a decrease in mortality between 3.1 and 12.1% (in the ICU admitted group), lower than the 12.6% of global mortality reduction (even higher in the ICU subset) that we found. We hypothesized that the effect of corticosteroid pulses might be higher than the low-dose corticosteroids because they act in different pathways (genomic vs. nongenomic) and behave, in fact, as different drugs [37]. Our study is not powered to compare both low-dose and pulse corticosteroid treatments, so we cannot assure this statement. Future studies must investigate this topic.

Pulse corticosteroids did not reduce the ICU admission rate in our study. Most patients were moved to the ICU in the first 24 hours of hospitalization (83.6%), so we understand that those patients were critically ill at admission time, requiring ICU in any case.

The in-hospital stay was significantly longer in the pulses corticosteroid arm, but those differences disappear in the survivor's group. Thus, this difference in the hospital stay was due to higher survival in the corticosteroid pulse group.

The rate of adverse events and serious adverse events declared was similar in both groups. In the same way, in-hospital infection and viral shedding time were similar in both groups, but some of these adverse events and infections might be underreported. The study was not powered to detect these adverse events because they were not always reported in the medical record in all patients.

In conclusion, this study provides evidence about the treatment with corticosteroid pulses in severe COVID-19 that might significantly reduce mortality. This data must be confirmed in prospective randomized studies.

## Figures and Tables

**Table 1:** Baseline features of patients from all centers combined.

Treatment	Overall population		Matched population	
	Methylprednisolone (n = 124)	Usual care (n = 133)	Methylprednisolone (n = 117)	Usual care (n = 88)
*Baseline characteristics*				
				
Gender (male) no- (%)	77 (62%)	69 (51.8%)	74 (63.3%)	45 (51.1%)
Age (mean-years)	74 [59-83]	76 [65-84]	75[60-83]	76 [66-83]
> 80 years no- (%)	37 (29.8%)	47 (35.3%)	36 (30.8%)	36 (30.8%)
Autoimmune disease	4 (3.2%)	3 (2.3%)	3 (2.5%)	3 (3.4%)
Cancer (%)	17 (13.7%)	17 (12.8%)	17 (14.5%)	11 (12.5%)
Chronic kidney disease (%)	6 (4.8%)	14 (10.5%)	6 (5.1%)	9 (10.2%)
Dementia (%) ∗	7 (5.6%)	21 (18.7%)	6 (5.1%)	12 (13.6%)
Diabetes	40 (32.3%)	52 (39.1%)	38 (32.5%)	36 (40.9%)
Dyslipidemia	44 (35.8%)	40 (30.1%)	42 (35.9%)	29 (33%)
Hypertension	63 (50.8%)	67 (50.4%)	42 (35.9%)	50 (56.8%)
Obesity	22 (17.8%)	18 (13.5%)	20 (17.1%)	13 (14.7%)
Smoker	32 (25.8%)	29(21.8%)	30(25.6%)	15 (17%)
Prior AIT/stroke (%)	7 (5.6%)	9 (6.8%)	7 (6%)	5 (5.7%)
Prior IHD (%)	12 (9.7%)	12 (9%)	12 (10.2%)	10 (11.4%)
Prior lung disease	20 (16.1%)	23 (17.3%)	20 (17.1%)	17 (19.3%)
Charlson comorbidity index	1 [0-2]	1 [0-4]	1 [0-2]	1 [0-2]

*Previous treatment*				
				
Corticosteroids	4 (3.2%)	2 (1.5%)	4 (3.4%)	1 (1.2%)
Anticoagulants	14 (11.2%)	10 (7.5%)	14 (11.9%)	6 (6.8%)

*Main findings at admission*				
				
Respiratory insufficiency	104 (83.9%)	105 (80.8%)	99 (84.6%)	73 (83.9%)
Bilateral infiltrates chest X-ray	103 (83.1%)	91 (68.4%)	105 (89.7%)	67 (76.1%)
pO2/FiO2	239.5 [142.2-276.1]	238.1 [192.8-285.7]	250 [153.2-276.2]	238 [180.5-276.1]
COVID GRAM	155.8 [83.3-230]	152.3 [69-235.6]	157.5 [83.6-231.6]	157.7 [82-233.2]

*Laboratory findings at admission*				
				
Glucose (mg/dl)	114.5 [94.2-145]	118.5 [100.1-168.2]	114.6 [94.5-147]	114.3 [99.2-180]
C-reactive protein (mg/L)	126.2 [84-200.2]	85.2 [36.1-180]	124.5 [66.7-194.5]	111.5 [60.9-213.5]
Creatinine (mg/dL)	1.06 [0.81-1.47]	0.99 [0.78-1.53]	1.06 [0.8-1.45]	1.01 [0.79-1.62]
D-dimer (ng/mL)^∗^	842 [448-1.450]	1,105 [540.2-2,532]	848 [4.127-1,425]	1,250 [678-2.532]
Ferritin (ng/mL)	1,533.5 [764-2,351.2]	921 [359.5-1,466]	1,475 [742-2,405]	1,104 [556-1,650]
Interleukin-6 (pg/mL)	35.5 [10.15-118.7]	51[17.4-119]	35.5 [10.1-118.7]	59 [17.6-123]
Lactate dehydrogenase (UI/L)	348.5 [283.7-460.7]	312[248-394]	341 [283-455]	326 [259-396.5]
Lymphocytes (cells/mm^3^)	1,000 [672-1,357]	1,000 [715-1,415]	1,000 [670-1,320]	1,010 [730-1,467]
Neutrophils (cells/mm^3^ × 10^3^)	5,050 [3,575-7,212]	5,670 [3,895-8,805]	5,997 [3,580-7,085]	5,155 [133-179]
Procalcitonin (ng/mL)	0.18 [0.1-0.37]	0.15 [0.07-0.5]	0.16 [0.1-0.3]	0.19 [0.1-1.5]

*Specific COVID-19 treatment*				
				
Azithromycin^∗^	115 (92.7%)	94 (71.2%)	108 (92.3%)	64 (73.3%)
Interferon beta-1b^∗^	48 (38.7%)	23 (17.4%)	44 (37.6%)	20 (23%)
Hydroxychloroquine^∗^	120 (96.8%)	121 (91.7%)	113 (96.6%)	80 (92%)
Lopinavir/ritonavir^∗^	106 (85.5%)	92 (69.7%)	102 (87.2%)	65 (74.7%)
Colchicine^∗^	16 (12.9%)	2 (1.5%)	15 (12.8%)	2 (2.3%)

*Nonspecific COVID-19 treatment*				
				
Prophylactic anticoagulation^∗^	69 (56.1%)	90 (68.7%)	66 (56.4%)	57 (66.3%)
Intermediate anticoagulation^∗^	12 (9.8%)	4 (3.1%)	10 (8.5%)	4 (4.7%)
Full anticoagulation^∗^	26 (21.1%)	16 (12.2%)	26 (22.2%)	13 (15.1%)

Data are shown as the median (IQR) or *n* (%). ^∗^Significant (*p* < 0, 05) difference between methylprednisolone and usual care population in both groups, the overall population, and propensity score match. It was calculated using the *χ*^2^ test or Kruskal-Wallis test as appropriate. PaO2/FiO2 = ratio of arterial oxygen partial pressure to fractional inspired oxygen.

**Table 2:** Primary and secondary outcomes of the global and matched population.

Outcome	Overall population			Matched population		
	Methylprednisolone (*n* = 124)	Usual care (*n* = 133)	*p* value	Methylprednisolone (*n* = 117)	Usual care (*n* = 88)	*p* value
*Primary outcome*						
						
60-day mortality	37/122 (30.3%)	57 (42.8%)	**0.026**	34/115 (29.6%)	39/88 (44.3%)	**0.022**

*Secondary outcomes*						
						
30-day mortality	37/122 (30.3%)	56/133 (42.1%)	**0.034**	34 (29%)	38 (43.2%)	**0.031**
Hyperglycemia	10 (8.0%)	5 (3.7%)	n.s	10 (8.5%)	5 (5.6%)	n.s
ICU admission	23 (18.5%)	26 (19.5%)	n.s	19 (16.2%)	21 (23.8%)	n.s
In-hospital mortality	36 (29%)	56 (42.1%)	**0,02**	33 (28.2%)	38 (43.2%)	**0.019**
LOS (days)	8 (10)	12 (13)		8 (11)	12 (12)	n.s
Mechanical ventilation	19 (15.3%)	25 (18.7%)	n.s	16 (13.6%)	21 (23.8%)	n.s
Nosocomial infection	29 (23.4%)	32 (24%)	n.s	26 (22.2%)	25 (28.4%)	n.s

Data are shown as the median (IQR) or *n* (%).ICU: intensive care unit; LOS: length of stay; n.s: not significant.

## Data Availability

We recorded all data in a database. These data could be requested if there is an important reason.

## Abbreviations

ARDS: Acute respiratory distress syndrome
COVID-19: Coronavirus disease 2019
ICU: Intensive care unit
PSM: Propensity score matching
SARS-CoV-2: Severe acute respiratory syndrome coronavirus 2
PCR: Polymerase chain reaction
IFN-*γ*: Interferon-*γ*
TNF-*α*: Tumor necrosis factor *α*
LDH: Lactate dehydrogenase
CRP: C-reactive protein.

## Additional Points

*Bullet Points*. (i) Corticosteroid pulses can improve mortality in severe COVID-19 patients. (ii) The Kaplan Meier curve with the propensity score matching and multivariate analysis in this group is reliable. (iii) There are no substantial changes in ICU admission and hospital stay.

## Supplementary Materials

Supplementary Materials Section A: Variable Description and Coding in the Study. Section B: Construction of the Propensity Score and Matching Process. Section C: Secondary Endpoints. Section D: Anticoagulation and Survival Analysis.
