# Supplementary material for: Corticosteroid Pulses for Hospitalized Patients with COVID-19: Effects on Mortality
Source: Mediators Inflamm. 2021 Mar 12;2021:6637227. doi: 10.1155/2021/6637227 (PMC7955656; doi:10.1155/2021/6637227)
Supplement: Supplementary Materials — Section A: Variable Description and Coding in the Study. Section B: Construction of the Propensity Score and Matching Process. Section C: Secondary Endpoints. Section D: Anticoagulation and Survival Analysis. [file 6637227.f1.docx]

**SUPPLEMENTARY MATERIAL:**

**Index:**

**SECTION A: VARIABLE DESCRIPTION AND CODING IN THE STUDY**

**SECTION B: CONSTRUCTION OF THE PROPENSITY SCORE AND MATCHING PROCESS**

**B1 Nearest neighbor method …………………………………………………. 8**

**B2 Exact matching method …………………………………………………….. 10**

**B3 Nearest neighbor with a caliper at 0.05 …………………………….. 11**

**B4 Nearest neighbor with a caliper at 0.1 ………………………………. 13**

**B5 Nearest neighbor with a caliper at 0.2 ………………………………. 15**

**B6 Nearest neighbor with a caliper at 0.25 …………………………….. 17**

**B7 Nearest neighbor with caliper at 0.3 …………………………………. 19**

**B8 Selection of the best method .…………………………………………… 21**

**SECTION C: SECONDARY ENDPOINTS**

**C1 30-days mortality ………………………………………………………………. 22**

**C2 Survival in ICU-admitted patients ………………………………………. 23**

**SECTION D: ANTICOAGULATION AND SURVIVAL**

**D1 Anticoagulation and survival analysis ………………………………… 25**

**SECTION A: VARIABLE DESCRIPTION AND CODING IN THE STUDY:**

- **Variables related to mortality:**
- X-Ray impairment
- Age
- Hemopyisis
- Low consciousness level
- SaO2 at inclusion
- FiO2 at inclusion
- Cancer
- Neutrophil / Lymphocyte index
- LDH
- Direct Bilirubin
- Number of comorbidities from 1 to 4 from these: chronic
- obstructive pulmonary disease, diabetes, hypertension, coronary
- artery disease, cerebrovascular disease, hepatitis B, cancer,
- chronic renal disease, immunodeficiency disease, and
- pregnancy.
- Modified Charlson index (Age, coronary artery disease, chronic heart failure, peripheral vascular disease, cerebrovascular disease, dementia, chronic obstructive pulmonary disease, connective tissue disease, peptic ulcer, liver disease, chronic kidney disease, diabetes, solid tumor, hemiplegia, leukemia, lymphoma, AIDS).
- COVID-GRAM calculation
- Sex
- Glucose
- Procalcitonin
- CRP
- IL-6
- D-Dimer
- Creatinin
- Ferritin
- Obese (Y/N)
- Epidemiologic week (week zero matches with the first death in Spain, March 3rd)
- Date of symptoms onset
- Stroke during hospitalization
- Myocardial infarction during hospitalization
- Pulmonary embolism during hospitalization
- Procalcitonin > 0.5 at any time
- Microbiological isolation (Y/N) and describe the isolation.
- Confirmed in-hospital infection (Y/N)
- Peak LDH
- Lowest Lymphocyte count
- Peak D-Dimer
- Peak ferritin
- Peak CRP

* Blood test data must be referred to the blood test in the ±3 days in which patients reach de inclusion criterion.

- **Concomitant treatments: (not allowed immunosuppressors)**
- Hydroxychloroquine
- Azithromycin
- Interferon-beta-1-b
- Lopinavir / Ritonavir
- Remdesivir
- **Inclusion / Exclusion criteria assessment:**
  - Date when PaFi decrease under 300, directly or indirectly measured:
  - PaO2
  - FiO2
- Pulse oxymetry when Pa/Fi decreased under 300 (estimated by non-linear imputation of SaFi to PaFi).

* PaFi <300 must be sustained more than 24 hours or repeated at least three times on different days. An isolated low value is not considered for inclusion.

* Values can be measured in the emergency department or during the hospitalization.

- Pregnancy
  - Age under 18
- Death in the first 24 hours: Admission date and death date
- Date of first SARS-Cov-2 PCR on the nasopharyngeal swab (or date of positive Elisa serology if compatible clinical features)
- Treatment with classic immunosuppressors (Y/N)
- Treatment with cytokine blockers like tocilizumab or anakinra (Y/N)
- Limitation of therapeutic effort or terminally ill patient at the admission days or within the first 24 hours of hospitalization (Y/N).
- On treatment with corticosteroids in a different way than described in inclusión criteria (Y/N)
- **Outcome related variables:**
- Admission date
- Discharge date
- Date of death (if applicable). Register if the patient died at 30 days once he was discharged, (Y/N).
- If the patient died: Cardiovascular death (Y/N). In the non-cardiovascular death subgroup register: Shock (1), refractory hypoxemia (3)0, multiorgan failure (3), Other (4). In the cardiovascular death subgroup register: Myocardial infarction(1), Arrhythmia (2), heart failure (3), cardiogenic shock (4), Other (5)
- Date of first negative SARS-Cov-2 PCR.
- Reach the definition of superinfection: PCT > 0.5
- ICU admission date
- The need for ventilatory support (invasive ventilation 1, non-invasive ventilation 2, no assistance needed 0)
- **Corticosteroid exposure:**

* Pulse corticosteroids are defined as 125-500 mg daily, for two to five days, within the ±3 days in which patients reach the inclusion criterion.

- Date onset of the first corticosteroid pulse
- The second course of corticosteroid pulses (Y/N)

**SECTION B: CONSTRUCTION OF THE PROPENSITY SCORE AND MATCHING PROCESS:**

The variables that have a statistical association in the univariate analysis between treatment with corticosteroid boluses (dichotomous 0 or 1) were the following:

- Center of Castilla y León (Hospital Clínico Universitario de Valladolid, Hospital Universitario de Salamanca, and Hospital Universitario de Burgos)

- The epidemiological week from the first positive PCR in early March

- Classic Charlson index

- Treatment with lopinavir/ritonavir at admission

- Treatment with azithromycin at admission

- Treatment with Interferon beta 1b at admission

- Treatment with colchicine on admission

- Need for non-invasive mechanical ventilation

- Presence of unilateral or bilateral radiological infiltrates

- Glucose at admission in mg/dl

- Ferritin at admission

- Ferritin or at admission or a week

- D-Dimer upon admission

- CRP upon admission

- D-Dimer upon admission or in the first week

- Dichotomous Charlson index (up to 2, and 3 or more comorbidities)

There is **no** statistical association between being treated with corticosteroids and the following variables:

- Age

- COVID-GRAM score (p-value between 0.05 and 0.1)

- Modified Charlson index

- Sex

- Hypertension

- Type 2 diabetes

- Dyslipidemia

- Smoker

- Obese

- Chronic ischemic heart disease

- Chronic kidney disease

- Significant lung disease

- History of cancer

- HIV

- Cerebrovascular disease

- Hemiplegia

- Previous rheumatological disease

- Moderate liver disease

- Presence of cirrhosis

- Peptic ulcer

- Liver disease of any severity

- Heart failure

- Dementia

- Peripheral artery disease

- Hemoptysis

- Low level of consciousness

- Previous treatment with oral anticoagulants

- Need for admission to ICU

- Need for invasive mechanical ventilation

- Presence of respiratory failure at the time of admission

- Degree of anticoagulation during admission

- Complications in the form of Pulmonary embolism, stroke, hemorrhage, or heart attack

- Neutrophils at admission and in the first week

- Lymphocytes at admission and in the first week

- Creatinine at admission and in the first week

- LDH at admission and in the first week

- CPK at admission and per week

- IL-6 levels both upon admission and the first week

- Direct bilirubin on admission

- Ferritin a week after admission

- D-Dimer one week after admission

- CRP and procalcitonin per week

- Maximum values ​​of LDH, ferritin, D-dimer, and CRP

- Minimum lymphocyte values

- Time from the onset of symptoms to admission

- Time from the onset of symptoms to PaFi less than 300

**Selected variables were dichotomized**, and the cutoff points selected for the non-dichotomous variables were the following:

- Blood glucose 162 mg / dl

- We opted for the dichotomized Charlson with a score or <2 or 2 or more than 2.

- Ferritin on admission or per week: 633

- CRP upon admission 95.55

- D-Dimer upon admission or per week: 1005

The qualitative variables of more than 2 categories are made dichotomous in this way:

Center: Hospital Clínico Universitario de Valladolid versus Hospital Universitario de Burgos or Hospital Universitario de Salamanca.

Epidemiological week: One group from weeks 0 to 2, and the other from week 3 onwards.

**Raw OR when doing binary logistic regression:**

Dichotomous epidemiological week: OR 3.8

Dichotomous center: OR 0.48

Glucose NS (neither cutoff point 160 nor 105 mg/dl)

Ferritin cutoff 633: OR 2.65

PCR cut 65.5: OR 2.28

D-dimer cut 1005 and cut 575: NS

Dichotomous Charlson: OR 0.5

Lopinavir/ritonavir treatment: 2.58

Azithromycin treatment: OR 5.22

Interferon beta 1b treatment: OR 3.12

Infiltrates in the Rx: OR 2.36

**The variables that remain in the final multivariate model are:**

Epidemiological week

Bilateral infiltrates or not

Center

Ferritin

Covid gram score

The final model has an area under the curve of 73.7%.

The distribution of the propensity score between those exposed and the not exposed ones to corticosteroids is this:


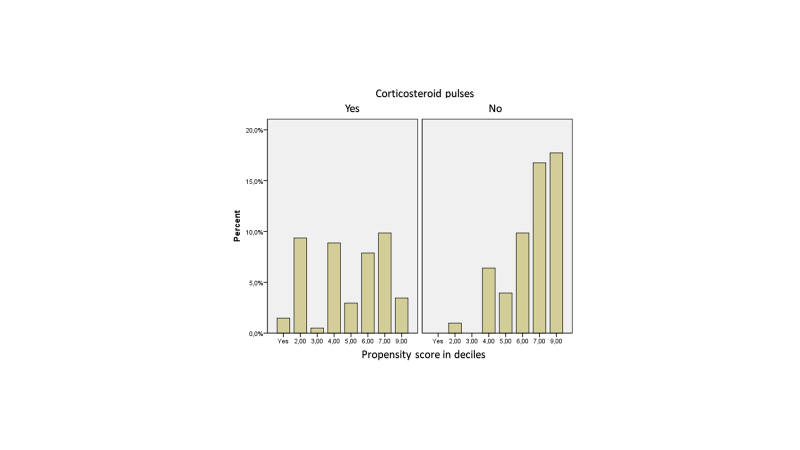


We propose 7 propensity score matching models using various mathematical methods. We compared the 7 models that a priori were better, by the following methods:

Nearest neighbor

Exact matching

Nearest neighbor con caliper de 0.05

Nearest neighbor con caliper de 0.1

Nearest neighbor con caliper de 0.2

Nearest neighbor con caliper de 0.25

Nearest neighbor con caliper de 0.30

We describe the results of the methods used for preprocessing

**B1 Nearest neighbor method**

**Love plot of the model:**

**Distribution of propensity scores in the model before and after matching:**

**B2 Exact matching method**

We do not show the raw and matched distribution because, in the exact method matching, all propensity scores are precisely the same in both groups.

**Love plot of the model:**

**B3 Nearest neighbor with a caliper at 0.05**

**Distribution of propensity scores in the model before and after matching:**

**Love plot of the model:**

**B4 Nearest neighbor with a caliper at 0.1**

**Distribution of propensity scores in the model before and after matching:**

**Love plot of the model:**

**B5 Nearest neighbor with a caliper at 0.2**

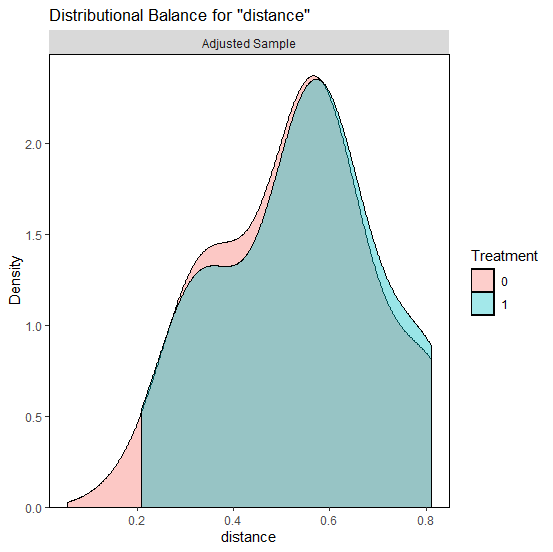


**Distribution of propensity scores in the model before and after matching:**


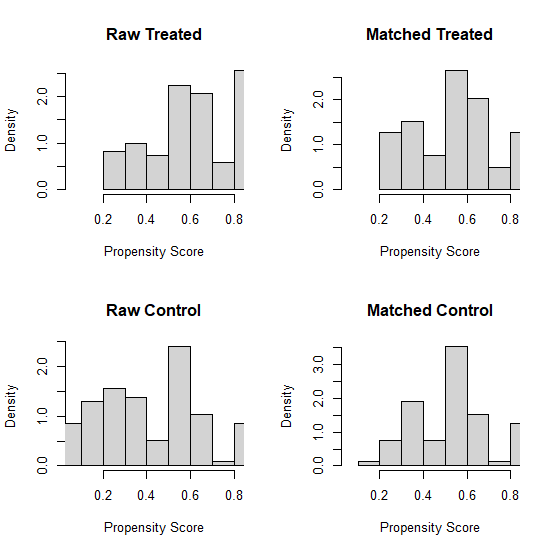


**Love plot of the model:**


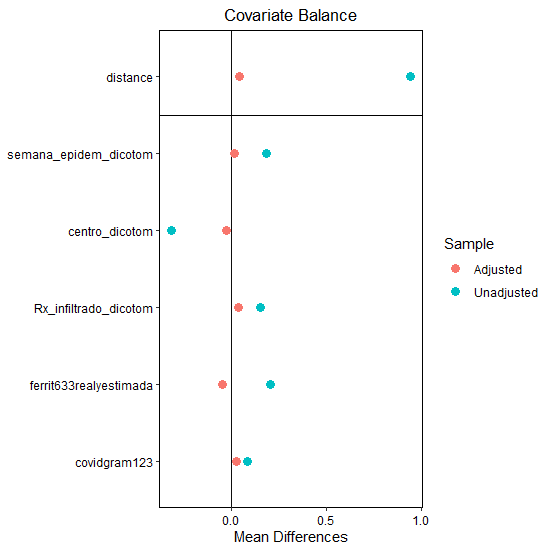


**B6 Nearest neighbor with a caliper at 0.25**

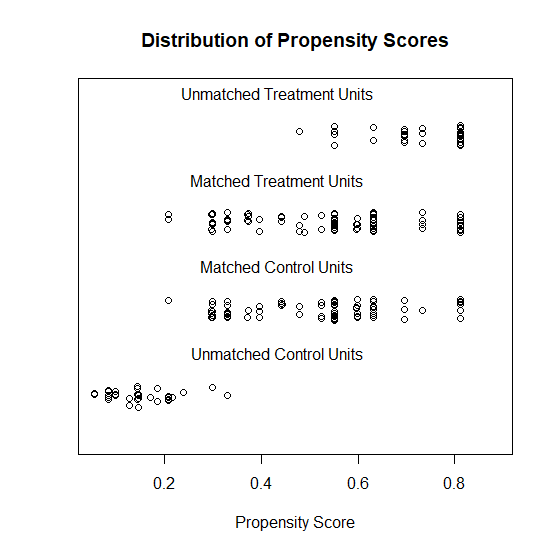


**Distribution of propensity scores in the model before and after matching:**


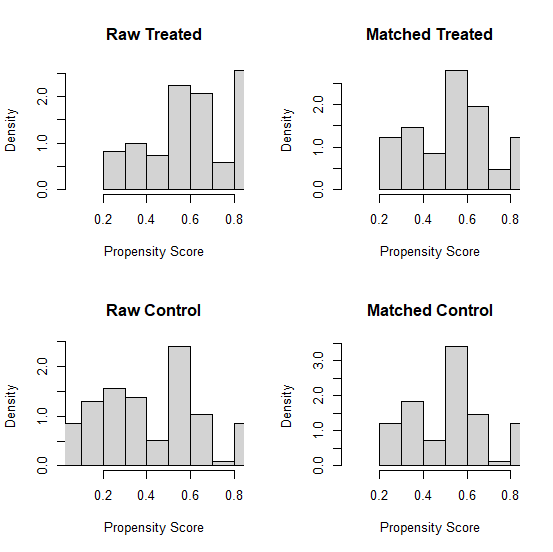


**Love plot of the model:**


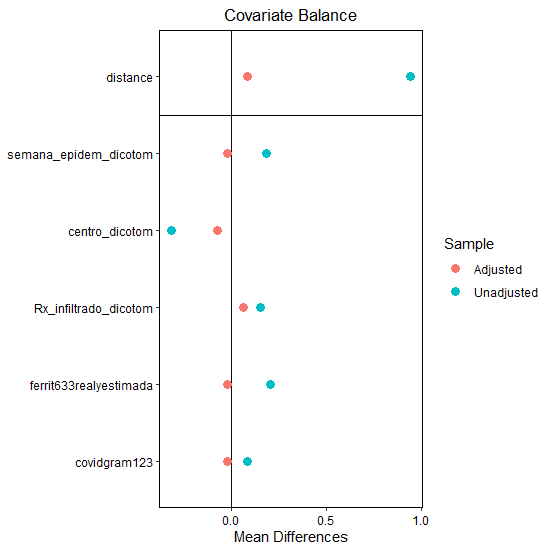


**B7 Nearest neighbor with caliper at 0.3:**

**Distribution of propensity scores in the model before and after matching:**


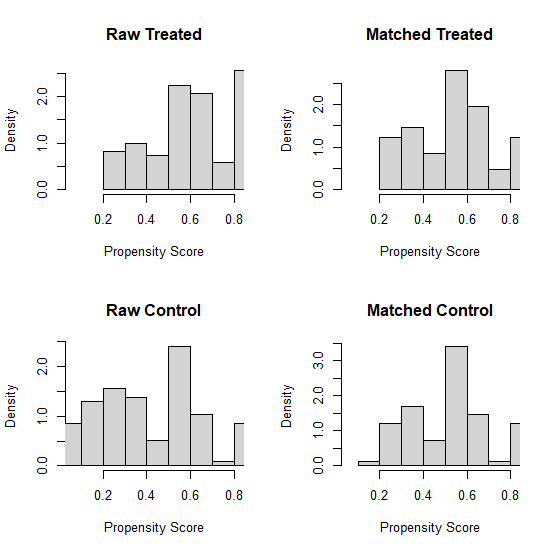


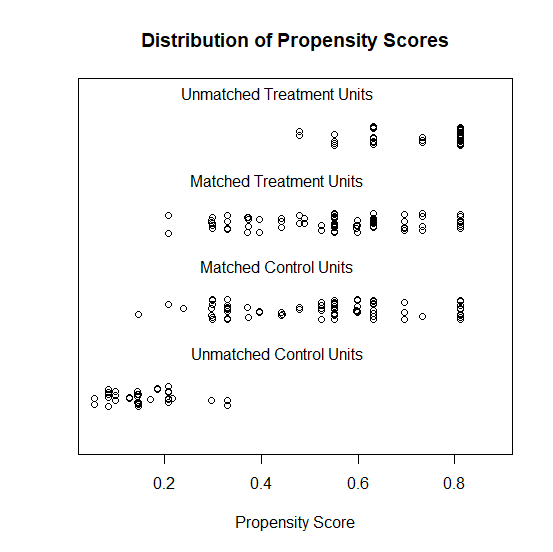


**Love plot of the model:**


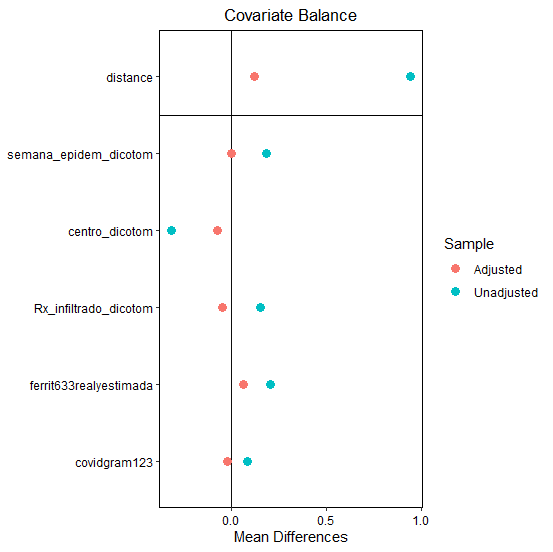


**B8 Selection of the best method**

We chose the **exact matching method** from all the available models because that is the one that loses the least number of patients with a better adjustment in terms of distance (propensity score) in those treated respecting controls.

**SECTION C: SECONDARY ENDPOINTS**

**C1. 30-days mortality**

**30-days mortality:** Kaplan Meier Curve in all participants and the propensity score matching group.


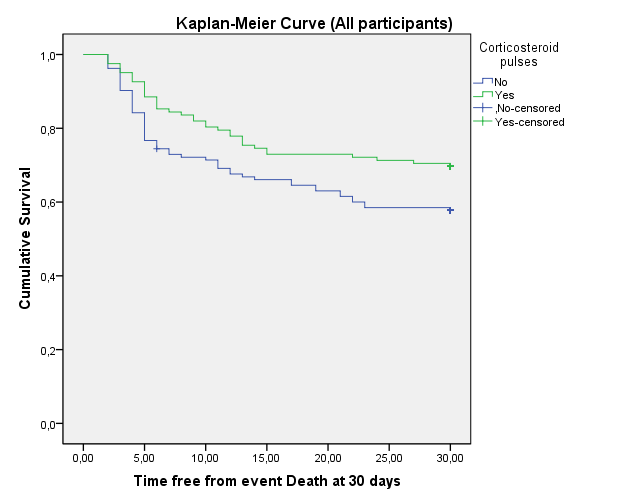


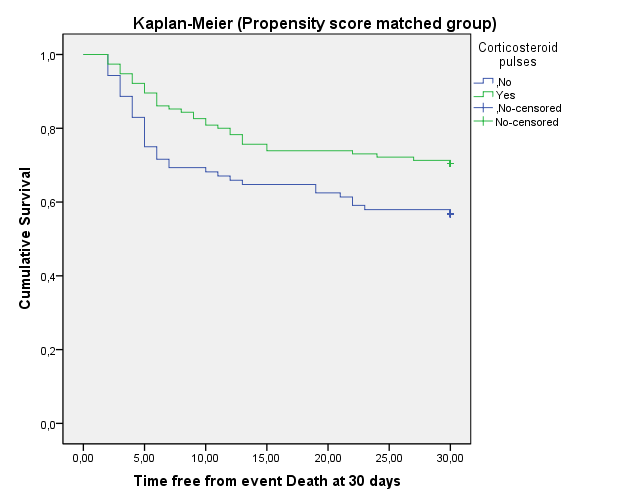


Cox regression model to predict 30-days mortality in the propensity score-matched group:

| **Variables in the Equation** | | | | | | | | |
| --- | --- | --- | --- | --- | --- | --- | --- | --- |
|  | B | SE | Wald | df | p-value | OR | 95,0% CI for OR | |
|  |  |  |  |  |  |  | Lower | Upper |
| Corticosteroid pulses | -,554 | ,281 | 3,877 | 1 | ,049 | ,575 | ,331 | ,997 |
| Age higher than 80 years | 2,048 | ,317 | 41,614 | 1 | ,000 | 7,753 | 4,161 | 14,445 |
| Neutrophil (Lymphocyte index higher than 7.4 | ,837 | ,294 | 8,117 | 1 | ,004 | 2,309 | 1,298 | 4,105 |
| Peak LDH > 372 UI/ml | ,726 | ,300 | 5,844 | 1 | ,016 | 2,067 | 1,147 | 3,725 |
| CRP > 200 mg/dl | 1,254 | ,309 | 16,515 | 1 | ,000 | 3,505 | 1,914 | 6,417 |
| Charlson index with 3 or more comorbidities | ,700 | ,300 | 5,441 | 1 | ,020 | 2,014 | 1,118 | 3,628 |

**C2. Survival in ICU-admitted patients:**

**
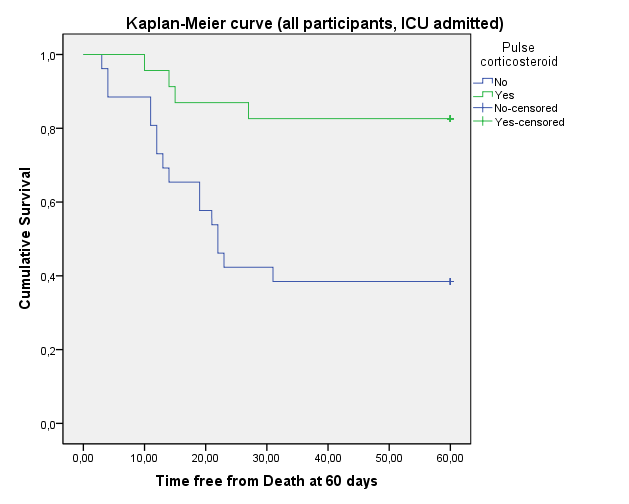
**

**
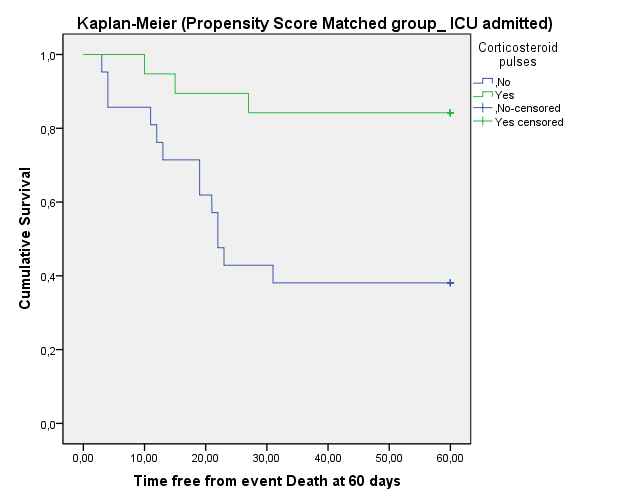
**

**SECTION D: ANTICOAGULATION AND SURVIVAL ANALYSIS**

**Survival analysis in patients with and without anticoagulation:**

**
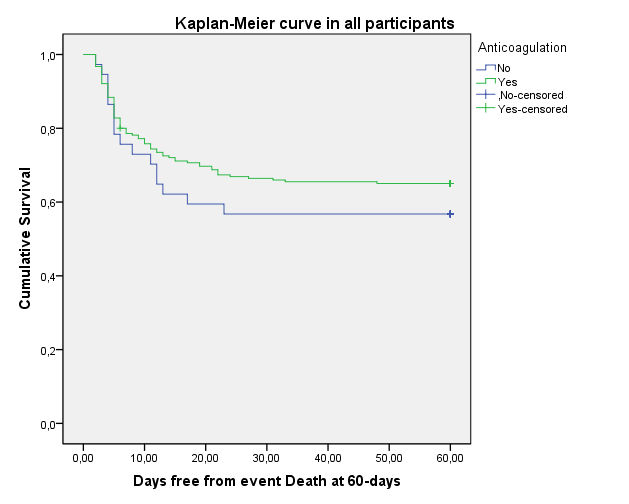
**

| **Overall Comparisons** | | | |
| --- | --- | --- | --- |
|  | Chi-Square | df | Sig. |
| Log Rank (Mantel-Cox) | ,918 | 1 | ,338 |
| Test of equality of survival distributions for the different levels of anticoagulation | | | |

| **Distribution of anticoagulation regimenes in patients with or without corticosteroid pulses** | | | | |
| --- | --- | --- | --- | --- |
| Count | | | | |
|  | | Corticosteroid Pulses | | Total |
|  |  | No | Yes |  |
| Heparin regimen | No anticoagulation | 21 | 16 | 37 |
|  | Prophilactic dose | 90 | 69 | 159 |
|  | Intermediate dose | 4 | 12 | 16 |
|  | Anticoagulant dose | 16 | 26 | 42 |
| Total | | 131 | 123 | 254 |

**Either the treatment with heparin or its intensity change survival in our study:**


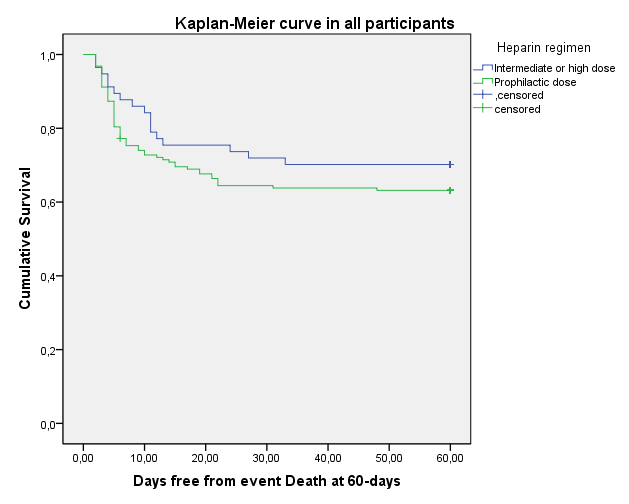


| **Overall Comparisons** | | | |
| --- | --- | --- | --- |
|  | Chi-Square | df | Sig. |
| Log Rank (Mantel-Cox) | 1,064 | 1 | ,302 |
| Test of equality of survival distributions | | | |
